# Genome-Wide Identification and Characterization of Long Noncoding RNAs of Brown to White Adipose Tissue Transformation in Goats

**DOI:** 10.3390/cells8080904

**Published:** 2019-08-15

**Authors:** Linjie Wang, Xin Yang, Yuehua Zhu, Siyuan Zhan, Zhe Chao, Tao Zhong, Jiazhong Guo, Yan Wang, Li Li, Hongping Zhang

**Affiliations:** 1 Farm Animal Genetic Resources Exploration and Innovation Key Laboratory of Sichuan Province, College of Animal Science and Technology, Sichuan Agricultural University, Chengdu 611130, Sichuan, China; 2 Institute of Animal Science and Veterinary Medicine, Hainan Academy of Agricultural Sciences, Haikou 571100, Hainan, China

**Keywords:** long noncoding RNA, perirenal fat, brown adipose tissue (BAT), goat

## Abstract

Long noncoding RNAs (lncRNAs) play an important role in the thermogenesis and energy storage of brown adipose tissue (BAT). However, knowledge of the cellular transition from BAT to white adipose tissue (WAT) and the potential role of lncRNAs in goat adipose tissue remains largely unknown. In this study, we analyzed the transformation from BAT to WAT using histological and uncoupling protein 1 (*UCP1*) gene analyses. Brown adipose tissue mainly existed within the goat perirenal fat at 1 day and there was obviously a transition from BAT to WAT from 1 day to 1 year. The RNA libraries constructed from the perirenal adipose tissues of 1 day, 30 days, and 1 year goats were sequenced. A total number of 21,232 lncRNAs from perirenal fat were identified, including 5393 intronic-lncRNAs and 3546 antisense-lncRNAs. Furthermore, a total of 548 differentially expressed lncRNAs were detected across three stages (fold change ≥ 2.0, false discovery rate (FDR) < 0.05), and six lncRNAs were validated by qPCR. Furthermore, *trans* analysis found lncRNAs that were transcribed close to 890 protein-coding genes. Additionally, a coexpression network suggested that 4519 lncRNAs and 5212 mRNAs were potentially in *trans*-regulatory relationships (*r* > 0.95 or *r* < −0.95). In addition, Gene ontology (GO) and Kyoto Encyclopedia of Genes and Genomes (KEGG) pathway enrichment analyses showed that the targeted genes were involved in the biosynthesis of unsaturated fatty acids, fatty acid elongation and metabolism, the citrate cycle, oxidative phosphorylation, the mitochondrial respiratory chain complex, and AMP-activated protein kinase (AMPK) signaling pathways. The present study provides a comprehensive catalog of lncRNAs involved in the transformation from BAT to WAT and provides insight into understanding the role of lncRNAs in goat brown adipogenesis.

## 1. Introduction

There are two class types of adipose tissue (white and brown) present in mammals. White adipose tissue (WAT) is used to store excess calories in the form of triglycerides and has an important role in the production of adipokines [1]. However, brown adipose tissue (BAT) is enriched with mitochondria and expresses high levels of uncoupling protein 1 (UCP1), which is localized on the inner membrane of mitochondria [2]. Uncoupling protein 1 is the primary marker of BAT. When activated, UCP1 stimulates adenosine triphosphate (ATP) synthesis and uncouples respiratory chain activity. Therefore, BAT plays an important role in heat production and nonshivering thermogenesis (NST) [3]. In recent studies, clusters of UCP1-expressing adipocytes called “beige” or “brite” [4] have been discovered in white adipose tissue (WAT) in response to cold stress and other stimuli [5,6,7]. In addition, beige adipocytes have thermogenic capacity as well as brown adipocytes [8].

The content of BAT is comparatively small and mainly exists in areas such as the perirenal, neck [9], supraclavicular, and pericardial regions of the human body [10,11]. The distribution of BAT is different between humans and rodents. In rodents, BAT is mainly clustered around the perirenal, interscapular, mediastinum, axillary, and cervical regions at birth. However, BAT is deposited in humans adults somewhere else: for example, BAT exists around the clavicular, neck, and paravertebral regions [12]. In sheep, BAT is recruited in perirenal adipose tissue at birth and then rapidly decreases at 30 postnatal days [13]. Furthermore, adult sheep retain BAT in both sternal and retroperitoneal fat, resulting in the conversion of stored energy into heat and nonshivering thermogenesis [14]. However, less is known about the cellular transition from BAT to WAT in goat perirenal adipose tissue.

Long noncoding RNAs (lncRNAs) over 200 bp in length are considered to have an important regulatory function. Previous studies have revealed that lncRNAs are associated with WAT and BAT development [15,16,17,18,19]. For example, brown fat lncRNA 1 (*Blnc1*) forms a transcriptional complex with early B cell factor 2 (EBF2) to promote brown adipogenesis [20]. Knockdown of AK079912, a novel brown adipose-enriched lncRNA, reduces lipid accumulation in brown adipocyte and downregulates BAT maker genes (*UCP1*, *PGC1α*, and *DIO2*) to inhibit brown adipogenesis [21]. Furthermore, lncBATE10 can interact with Celf1 (RNA-binding protein) to regulate brown adipocyte differentiation [22]. In addition, lncBATE1 sustains the core brown adipose marker genes program and represses the expression of white adipose marker genes. Additionally, lncBATE1 interacts with the nuclear matrix factor (hnRNP U) to promote brown adipogenesis [23]. These findings indicate important roles for lncRNAs during WAT browning in human and mouse models.

Despite this recent progress, knowledge of the cellular transition from BAT to WAT and the potential role of lncRNAs in goat brown adipose tissue remains largely unknown because lncRNAs are poorly conserved among different species. Thus, before the regulatory function of lncRNAs in goat adipose can be revealed, it is necessary to provide a comprehensive catalog of lncRNAs of the transformation from goat BAT to WAT. In this study, we identified the differentially expressed lncRNA molecules of the transformation from BAT to WAT during perirenal fat development and provide insight into understanding the role of lncRNAs in goat brown adipogenesis.

## 2. Material and Methods

### 2.1. Ethics Statement

All research involving animals was conducted according to the Regulations for the Administration of Affairs Concerning Experimental Animals (Ministry of Science and Technology, China, revised in June 2004) and was approved by the Institutional Animal Care and Use Committee at the College of Animal Science and Technology, Sichuan Agricultural University, Sichuan, China, under permit “No. DKY-B20110807”.

### 2.2. Animal and Sample Collection

The Chuanzhong black goats used in this study were raised at the breeding center of the Sichuan Agricultural University, Ya’an, China. These ewes were fed a standard diet (forage to concentrate ratio, 70:30) twice per day at 07:00–09:00 and 16:00–18:00 and water ad libitum. Perirenal adipose tissues were collected from 12 female goats at 3 different postnatal stages (1 day, 30 days, and 1 year after birth of the goat, 4 individuals at each stage, denoted as D1, D30, and Y1, respectively). Perirenal adipose tissues were dissected immediately, snap frozen in liquid nitrogen, and stored at −80 °C for sequencing analysis.

### 2.3. Histology Analysis

The samples of perirenal fat were fixed in 4% paraformaldehyde at room temperature and embedded by paraffin. Three 3-μm sections were stained with hematoxylin. Pictures of 3 consecutive 20× power fields were captured with an Olympus BX-50F light microscope (Olympus Optical, Tokyo, Japan). Image analysis software (Case Viewer, Budapest, Hungary) was applied to select areas of adipocytes.

### 2.4. Immunohistochemistry

Immunohistochemistry was undertaken on perirenal adipose tissues from three different postnatal stages. Adipose tissues were embedded in optimal cutting temperature (OCT) compound (Miles, Elkhart, IN, USA), snap frozen, and stored at −80 °C. Sections 5 μm thick were cut with a cryostat (Leica, Bensheim, Germany) and then incubated overnight at 4 °C with the primary antibody (anti-uncoupling protein 1 (UCP1), 1:500, Abcam, California, USA). After phosphate buffer saline (PBS) washing for 20 min, the secondary antibody (horseradish peroxidase-conjugated goat antirabbit IgG, 1:2000) was incubated for 1 h. The streptavidin-biotin complex (SABC) and 3,3′-Diaminobenzidine tetrahydrochloride (DAB) visualization methods were used according to the manufacturer’s instructions (Boster Company, Wuhan, China). Stained sections were captured using an Olympus BX-50F light microscope (Olympus Optical, Tokyo, Japan).

### 2.5. RNA Extraction, Library Construction, and Sequencing

Total RNA was extracted from adipose tissues using TRIzol (Invitrogen, CA, USA). The RNA amount and purity were quantified using a NanoDrop 2000 spectrophotometer (Thermo Fisher Scientific, Wilmington, DE, USA). Moreover, RNA integrity was assessed by an RNA Nano 6000 Assay Kit of the Agilent 2100 bioanalyzer (Agilent Technologies, CA, USA). The NEBNextR UltraTM RNA Library Prep Kit (NEB, MA, USA) was used to generate the sequencing library according to the manufacturer’s recommendation. Finally, the library preparations were sequenced by the Hiseq4000 platform (Illumina, San Diego, CA, USA), and paired terminal readings were generated. Data are available at the Sequence Read Archive (SRA) database, accession number PRJNA547456.

### 2.6. Quality Control and Transcriptome Assembly

Raw reads in FASTA format were primarily handled by in-house Perl scripts. At this stage, clean reads were acquired by removing reads that contained an adapter, were of low quality, or had over 10% of ploy-N (undefined base) reads from raw reads. Then, HISAT2 [24] was used to align clean reads to the goat reference genome (https://www.ncbi.nlm.nih.gov/genome/?term=goat) [25]. StringTie (v1.3.1) [26] was used to assemble the transcriptome based on the reads mapped to the goat reference genome.

### 2.7. Identification and Expression Analysis of Potential lncRNA

First, potential lncRNAs from transcripts should be more than 200 nt and have more than one exon [27]. Second, the fragments per kb per million reads (FPKM) score should be more than 0.5. Third, the transcripts should have no coding potential. Four tools, the Coding Potential Calculator (CPC), the Coding Non Coding Index (CNCI), the Protein Families Database (Pfam), and the Coding Potential Assessment Tool (CPAT) [28,29,30,31], were used to assess the protein-coding potential of the assembled transcripts. The expression level of lncRNAs was normalized using the FPKM score, and Cuffdiff (v2.1.1) [32] software was used to calculate FPKM scores in each library. Differential expression analysis was performed using the DESeq package [33]. The differentially expressed lncRNAs with a false discovery rate (FDR) < 0.05 and |log2(FoldChange)| ≥ 1 were assigned as significantly differential expressions.

### 2.8. Target Gene Prediction and Enrichment Analysis

To predict the *cis*-regulated target genes, we searched the potential protein-coding genes 100 kb upstream and downstream of differentially expressed lncRNAs using LcnTar software [34]. To predict the *trans*-regulated target genes, coexpression analysis was calculated between the expression levels of lncRNAs and protein-coding genes based on a Pearson correlation ≥0.95 or ≤−0.95. Gene ontology enrichment analysis of target genes was performed by the GOseq R package [35]. In addition, KOBAS software 2.0 [36] was used to determine the enrichment of target genes in KEGG pathways (http://www.genome.jp/kegg/). The significantly enriched GO terms and KEGG pathways were determined using *p* < 0.05 as thresholds.

### 2.9. Quantitative Real-Time PCR

To validate the differentially expressed lncRNAs in RNA-seq and *UCP1* gene expression, qPCR was used to quantify the expression level of lncRNAs and UCP1 in three stages. Each qPCR (in 10 μL) reaction system included 5 μL TB Green Premix Ex TaqП (Takara, Tokyo, Japan), 0.4 μL primers (Table S8), and 0.5 μL cDNA. The cycling conditions were formed by a single cycle for 3 min at 95 °C, followed by 40 cycles of circulation of 20 s at 94 °C, 20 s at 62.3 °C–63.3 °C, 15 s at 72 °C, and final extension for 5 min. The cDNA was diluted to a standard curve to confirm the efficiency of the reaction system. The reference gene for the gene expression level applied β-actin to calculate *Ct (2^−ΔΔCt^)*.

## 3. Results

### 3.1. Characterization of the Transformation from BAT to WAT during Perirenal Fat Development

**Figure 1:**
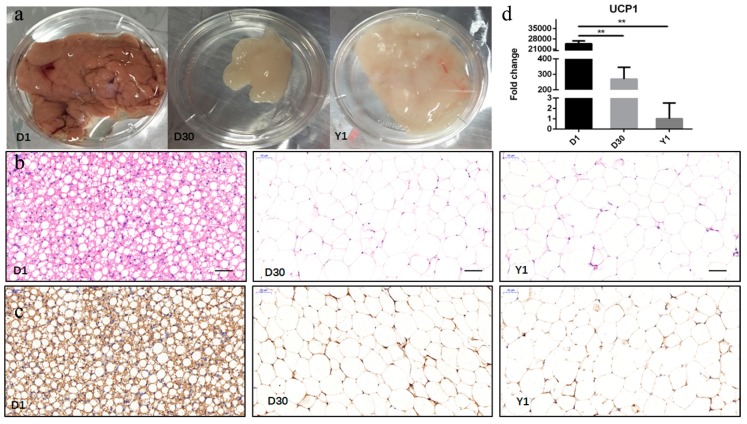
Characterization of transformation from brown adipose tissue (BAT) to white adipose tissue (WAT) during perirenal fat development. (**a**) Representative images are shown for goat perirenal fat 1 day (D1), 30 days (D30), and 1 year (Y1) after birth of the goat. (**b**) Histological sections stained with hematoxylin (HE). (**c**) Uncoupling protein 1 (UCP1) immunostaining from perirenal adipose tissue at three stages. Scale bars: 50 μm. (**d**) Changes in mRNA expression levels of UCP1. Error bars represent standard error of mean (SEM), *n* = 4, ** *p* < 0.01.

To evaluate the histological diversity of perirenal fat in three stages, a histological analysis was performed. From D1 to Y1, there was an obvious transition in the histological appearance of the perirenal fat (Figure 1a). In the hematoxylin-eosin (HE)-stained adipose section, BAT was a multichamber structure, and lipid droplets of BAT were smaller than WAT. In addition, adipocytes displayed unilocular morphology at D30 and Y1 (Figure 1b).

To further determine the transformation from brown to white adipose tissue, we detected the content of UCP1 in abundance at different stages of perirenal fat development by immunohistochemistry and qPCR. There was a notable maximal UCP1 content at birth. Throughout the postnatal period, the content of UCP1 decreased, with the gradual disappearance of BAT (Figure 1c). Subsequently, qPCR results indicated that the expression levels of *UCP1* were the highest at D1, then greatly (*p* < 0.01) decreased at D30, reaching the lowest expression level at Y1 (Figure 1d). The results demonstrated that BAT mainly existed within the perirenal fat at D1, and there was obvious transition from BAT to WAT from D1 to Y1.

### 3.2. Identification and Characterization of lncRNA in Perirenal Fat at Three Stages

**Figure 2:**
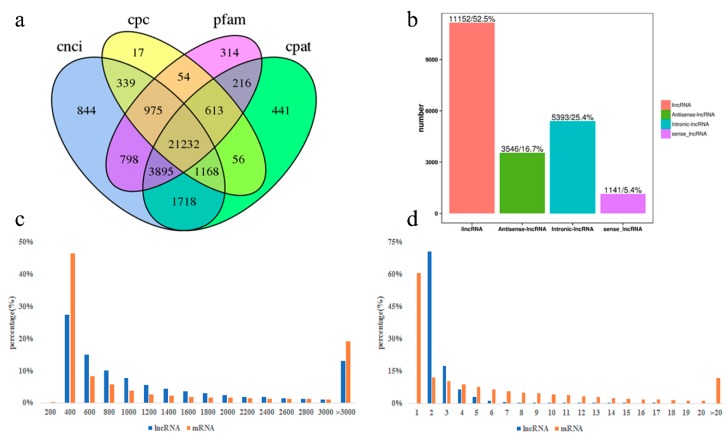
Comparison of the features of goat long noncoding RNAs (lncRNAs) and mRNA. (**a**) Venn diagram showing the predicted lncRNAs with the four computational approaches. (**b**) Type and number of predicted lncRNAs. (**c**) Length per transcript of goat lncRNAs and mRNA. (**d**) Exon numbers per transcript of goat lncRNAs and mRNA.

To identify lncRNAs expressed in goat perirenal fat at three stages, we sequenced the total RNA from samples of three stages using the Illumina HiSeq4000 platform. A total amount of 1,429,936,306 paired-end clean reads were produced from the 12 cDNA libraries (213.45 Gb), and 95.75%–96.96% of the clean reads (Table S1) were mapped to the goat reference genome. After analysis using CNCI/CPC/pfam/CPAT software, 21,232 lncRNAs were identified (Figure 2a), including 11,152 lincRNA (52.5%), 5393 intronic-lncRNA (25.4%), 3546 antisense-lncRNA (16.7%), and 1141 sense-lncRNA (5.4%) (Figure 2b and Table S2). A total number of 21,232 lncRNAs were expressed in goat perirenal fat, with a median length of 737 bp and 2.51 exons, which was shorter than the mRNA genes with an average length of 2035 bp and 4.16 exons (Figure 2c,d).

### 3.3. Identification of Differentially Expressed lncRNAs during the Transformation from Brown to White Adipose Tissue

**Figure 3:**
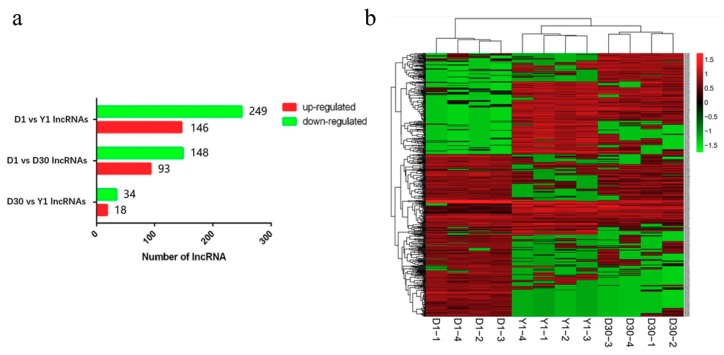
Analyses of differentially expressed lncRNAs from 12 libraries. (**a**) Numbers of upregulated and downregulated lncRNAs in three stages of perirenal fat. (**b)** Hierarchical cluster analysis of differently expressed lncRNAs in perirenal fat of goats. The red signal refers to relatively high expression, and the green signal refers to relatively low expression.

The expression levels of lncRNAs during the transformation from goat brown to white adipose tissue were estimated using FPKM values. Through pairwise comparison of differentially expressed lncRNAs, 548 differentially expressed lncRNAs (fold change ≥ 2.0, FDR < 0.05) were detected across three stages (Table S3). The highest number of differentially expressed lncRNAs were found between D1 and Y1, including 146 upregulated and 249 downregulated lncRNAs. Between D1 and D30, 93 lncRNAs were upregulated and 148 lncRNAs were downregulated. There were only 18 upregulated and 34 downregulated lncRNAs between D30 and Y1 (Figure 3a). Next, to determine the similarities and the relationships between the three stages, 548 differentially expressed lncRNAs were used to perform a clustering analysis. As shown in Figure 3b, the four replicates for each stage clustered together. In addition, the samples from three periods were divided into two clusters, and D1 formed one cluster, and D30 and Y1 formed another cluster.

### 3.4. The Cis-Regulated Target Genes of lncRNAs

**Figure 4:**
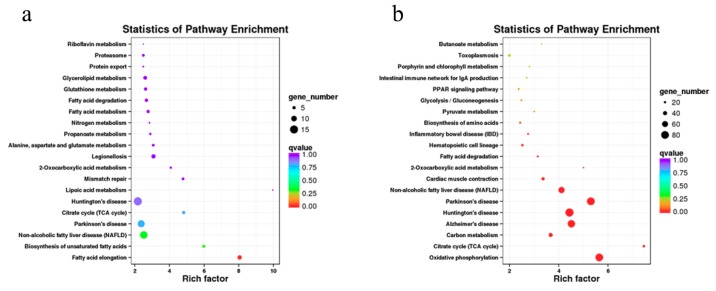
KEGG pathway map of differently expressed lncRNAs in perirenal fat of goats. Top 20 significantly enriched KEGG pathways for *cis*- (**a**) and *trans*-regulated (**b**) target genes of lncRNAs. Each scatter point represents a pathway. The size of each point represents the degree of enrichment, and the color of each point represents the size of the *q*-value. The *q*-value represents multiple hypothesis testing using the Benjamin–Hochberg procedure [37].

To search the *cis*-regulated target genes, we predicted the potential protein-coding genes of 548 differentially expressed lncRNAs using 100 kb upstream and downstream as the cutoff. Our results included 330 lncRNAs that were transcribed close to 890 protein-coding genes (Table S4). The *cis*-lncRNA target genes were annotated and were mainly enriched with the following: G-protein-coupled receptor activity, a mitochondrion, protein kinase activity, receptor binding, and a ribosomal subunit (Table S5). Meanwhile, KEGG enrichment analysis indicated that the 890 candidate *cis* target genes were significantly enriched in 22 signaling pathways (Table S6). The top 20 KEGG pathways are shown in Figure 4a, including fatty acid elongation, the biosynthesis of unsaturated fatty acids, nonalcoholic fatty liver disease (NAFLD), fatty acid degradation, fatty acid metabolism, oxidative phosphorylation, the citrate cycle (TCA cycle), and the AMPK signaling pathway.

### 3.5. Enrichment Analysis of Coexpressed Genes of lncRNAs

We also observed the potential *trans*-regulated target genes of lncRNAs using coexpression analysis. A total of 3075 pairs were obtained between 4519 lncRNAs and 5212 protein-coding genes based on a Pearson correlation ≥0.95 or ≤−0.95 (a Table S7). A functional analysis indicated that the coexpressed genes were significantly enriched in 192 GO terms, including G-protein-coupled receptor activity, the electron transport chain, the respiratory chain, the mitochondrial respiratory chain complex, the mitochondrial matrix, cytochrome-c oxidase activity, nicotinamide adenine dinucleotide (NADH) dehydrogenase (ubiquinone) activity, and ATP synthesis coupled proton transport (Table S5). In addition, the coexpressed genes were significantly enriched in 58 KEGG pathways, including oxidative phosphorylation, the citrate cycle (TCA cycle), fatty acid degradation, fatty acid elongation, fatty acid metabolism, nonalcoholic fatty liver disease (NAFLD), chemokines, and the peroxisome proliferators-activated receptor (PPAR) signaling pathway (Figure 4b and Table S6).

### 3.6. Validation of lncRNAs by qPCR

**Figure 5:**
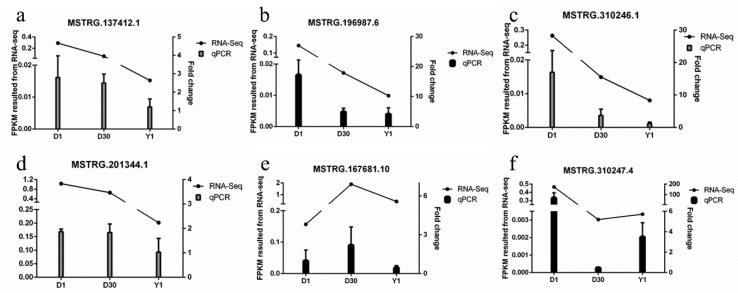
Validation of six differentially expressed lncRNAs ((**a**), MSTRG.137412.1; (**b**), MSTRG.196987.6; (**c**), MSTRG.310246.1; (**d**), MSTRG.201344.1; (**e**), MSTRG.167681.10; (**f**), MSTRG.310247.4) by qPCR. Left *y* axis (line chart) shows the fragments per kb per million reads (FPKM) values of the lncRNAs using RNA-seq, and the right *y* axis (bar chart) shows the relative expression levels of lncRNAs using qPCR. Data are shown as means ± SEM. Four biological replicates were used.

Furthermore, we randomly selected six lncRNAs for validation using qPCR analysis. As shown in Figure 5a–d, the expression levels of lncRNAs MSTRG.137412.1, MSTRG.196987.6, MSTRG.310246.1, and MSTRG.201344.1 were downregulated from D1 to Y1. MSTRG.167681.10 was at the lowest level in D1 and then increased to a peak in D30 and was downregulated from D30 to Y1 (Figure 5e). In addition, MSTRG.310247.4 was expressed at the highest level at D1 and decreased to the lowest level at D30, then was upregulated at Y1 (Figure 5f). These results indicated that the entire qPCR expression tendency was consistent with the sequencing results, implying that the expression patterns of lncRNA at three stages using RNA-seq were reliable.

## 4. Discussion

Many studies have demonstrated that *UCP1* expression varies obviously during micromorphology and transcription changes in infant and rodent perirenal fat [12,38,39]. In sheep, the perirenal adipose tissue comprises mainly brown adipocytes from day 0 to day 4 after birth, whereas white adipose tissues are predominant from day 14 after birth, according to histological confirmation. Besides, there is a maximum *UCP1* content at birth, and then the expression of *UCP1* decreases to the lowest level at 30 d [13]. One study focused on the early postnatal period, but did not extend it to the period from birth to adulthood in perirenal fat. In our study, the postnatal transformation from goat BAT to WAT occurred within 1 year after birth as determined by changes in tissue morphology and *UCP1* gene expression. We showed that BAT was recruited in goat perirenal adipose tissue at birth, whereas WAT was predominant from D30 to Y1 in a histological analysis. In addition, the expression of *UCP1* was at the highest level at D1, and then significantly decreased at D30, reaching its lowest expression at Y1. These results suggest that BAT mainly existed within the perirenal fat at D1, and there was obvious transition from BAT to WAT during goat perirenal fat development.

A previous study reported that many lncRNAs have been identified from adipocytes and are induced during adipogenesis [40]. In recent studies, the number of lncRNAs has been discovered in the development of BAT, such as in humans [41] and mice [23]. A previous study of sheep tail adipose tissues showed that there are many differentially expressed lncRNAs among three breeds [42]. Here, we identified a total of 21,232 lncRNAs across three development stages from perirenal fat in D1, D30, and Y1 goats. The numbers of lncRNAs in the goat perirenal fat were higher than those reported in goat skin (1108 lncRNAs) [43], the hypothalamus (2943 lncRNAs) [44], and skeletal muscle (3981 lncRNAs) [45]. Previous studies reported that lncRNAs are strongly tissue-specific compared to protein-coding genes in different human tissues [46]. A large number of adipose tissue-specific lncRNAs have been identified from mouse brown and white adipose tissues [23]. Thus, the lncRNAs observed in the present study may be largely adipose tissue-specific and may have specific functions in the transformation from goat BAT to WAT.

The identified lncRNAs had fewer exon numbers, shorter lengths, and lower conservation and expression levels than protein-coding genes. The median length of goat lncRNAs (737 bp) was longer than that of human lncRNAs (592 bp) [47]. We infer that these different results might have been caused by the exclusion of single-exon lncRNAs. Illumina sequencing cannot distinguish short single exons from transcriptional noise. During the process of RNA-seq data processing, single exon transcripts are generally filtered out, because the single-exon transcript assembly sequence has low reliability [27,48]. In this study, we provided a comprehensive catalog of lncRNAs of the transformation from BAT to WAT. Furthermore, we built a regulatory network by analyzing coexpression patterns of lncRNAs and mRNAs, suggesting their functional associations in the transformation from BAT to WAT. Our work serves as a resource to study the regulatory function of lncRNAs in the transformation from BAT to WAT.

Then, we predicted the potential target genes of 548 differentially expressed lncRNAs, which can function in *cis* and *trans*. The *cis*-lncRNAs usually form a feedback loop that regulates itself and the neighboring genes, thus acting on neighboring genes on the same allele that it transcribes [49]. Fatty acid metabolism, fatty acid elongation, and the biosynthesis of unsaturated fatty acids were significantly enriched by many target genes of lncRNAs. The biosynthesis of unsaturated fatty acids is essential for adipose tissue development and is a key process in the synthesis of long-chain unsaturated fatty acids [50], fat synthesis and biohydrogenation [51], the effects of *trans*-fatty acids [52], and fatty liver disease regulation [53,54]. Fatty acid elongation and metabolism pathways regulate multiple physiological functions, including the synthesis of long-chain fatty acids, body weight and fat accumulation [55], the activation of *UCP1* in brown-fat mitochondria, and the metabolism of adipocyte energy [56,57].

In the *cis* prediction, we found that some of the *cis* target protein-coding genes (*HADHA*, *HADHB*, *CPT1A*, *SDHD*, and *ECHS1*) were involved in these pathways, implying that the corresponding lncRNAs play regulatory roles in fatty acid metabolism. For example, the *HADHA* and *HADHB* genes encode the α- and β-subunits of the mitochondrial trifunctional protein, respectively, which affect the mitochondrial β-oxidation activity of long-chain fatty acids [58,59]. Further, *CPT1A* is the key enzyme of fatty acid β-oxidation in the mitochondria, and *CPT1A* knockout reduces glucagon secretion and fatty acid β-oxidation in pancreatic islet α-cells [60]. In a recent study, hepatic *CPT1A* was directly activated by baicalin to promote the lipid going into mitochondria for fatty acid degradation, which could ameliorate hepatic steatosis and obesity [61]. In particular, *HADHA*, *HADHB*, *CPT1A*, and *ECHS1* were regulated by several lncRNAs in *cis*. It is worth noting that many target genes were significantly enriched in the AMPK signaling pathway, which is a critical pathway for energy metabolism [62] and the browning of white adipocytes [63]. In addition, AMPK α1- and α2-subunit knockout reduced adipogenesis and thermogenic function in brown adipose tissue compared to wild-type mice [64,65]. In adipose tissue-specific knockout of AMPK β1- and β2-subunit mice, AMPK was essential for mitochondrial integrity and thermogenesis upon cold and β-adrenergic stimulation and promoted the browning of white adipose tissue [66].

The coexpression analysis indicated that most regulated genes were mainly enriched in the citrate cycle (TCA cycle) and the oxidative phosphorylation pathway. The most commonly enriched regulated genes were the *ATP5*, *SDHA*, *SDHB*, *SDHD*, *NDUF*, and *IDH3* family genes. The *ATP5* family genes encode different subunits of mitochondrial ATP synthase, which exists in the inner membrane of mitochondria and produces adenosine triphosphate (ATP) from adenosine diphosphate (ADP) during oxidative phosphorylation [67]. Succinate dehydrogenase (SDH) is a key enzyme of the TCA cycle and is a part of complex II in the mitochondrial electron transport chain. In addition, succinate dehydrogenase plays a key role in the transfer of electrons from succinate to ubiquinone [68]. In addition, there are many target genes encoding the subunits of NADH dehydrogenase, which is the center of complex I, embedded in the inner mitochondrial membrane. The transferring of electrons from nicotinamide adenine dinucleotide (NADH) to coenzyme Q (CoQ) is regulated by NADH dehydrogenase [69,70]. Interestingly, UCP1 is the primary marker of BAT and plays an important role in ATP synthesis and uncouples respiratory chain activity in BAT [3]. Using the coexpression analysis, we found that the *UCP1* gene was regulated by several lncRNAs, including MSTRG.195615.2, MSTRG.211247.2, MSTRG.223440.2, MSTRG.22947.2, and MSTRG.230678.1. These results indicate that these lncRNAs may be involved in the transformation from BAT to WAT through regulating their target gene *UCP1*. It is suggested that the biological pathways regulated by the identified lncRNAs are directly linked to the mitochondrial oxidative phosphorylation and ATP synthesis of the transformation from BAT to WAT. Finally, there were some limitations to our research. Although we identified some pathways that are involved in the transformation from BAT to WAT, the molecular mechanisms of these lncRNAs in the function of BAT still need to be investigated in future research.

## 5. Conclusions

In this study, we provided a comprehensive catalog of lncRNAs in the transformation from BAT to WAT. A total number of 21,232 lncRNAs were identified from goat perirenal fat, of which 548 differentially expressed lncRNAs were detected during the transformation from BAT to WAT. Furthermore, the coexpression analysis of lncRNAs and mRNAs revealed that the targeted genes were largely involved in fatty acid metabolism, oxidative phosphorylation, the mitochondrial respiratory chain complex, and the AMPK signaling pathways, suggesting that lncRNAs regulate the transformation from BAT to WAT, possibly through regulating genes involved in the above signaling pathway. This study provides evidence for the role of lncRNAs in the transformation from BAT to WAT in goats and is a basis for greatly improving the annotation of the goat reference genome.

## Supplementary Materials

The following are available online at https://www.mdpi.com/2073-4409/8/8/904/s1. Table S1: Summary of RNA-Seq data and reads mapped to the goat genome. Table S2: List of lncRNAs identified in the goat perirenal fat libraries. Table S3: List of DEG lncRNAs from three developmental stages in perirenal fat. Table S4: The protein-coding genes within 100-kb upstream and downstream of the differentially expressed lncRNAs. Table S5: Functional enrichment analysis of trans-regulated target genes of lncRNAs. Table S6: The information of KEGG pathway of trans-regulated target genes of lncRNAs. Table S7: Co-expression analysis between protein coding genes and lncRNAs. Table S8: Sequences of qPCR primers for lncRNAs and UCP1.
