# Supplementary material for: Genome-Wide Identification and Characterization of Long Noncoding RNAs of Brown to White Adipose Tissue Transformation in Goats
Source: Cells. 2019 Aug 15;8(8):904. doi: 10.3390/cells8080904 (PMC6721666; doi:10.3390/cells8080904)
Supplement: Supplementary file 1 [file cells-08-00904-s001.zip › Table S8.pdf]

Sequences of qPCR primers for lncRNAs and UCP1

Genes	Accession numbers	Sense	Anti-Sense	Optimum temperature	Amplicon length (bp)
MSTRG.137412.1	NC_030817.1	TCTGGGTTTTAGCTAGCATGGA	GTCGAAGAAGGCACAAGCAA	56.9℃	196
MSTRG.167681.10	NC_030819.1	GATCGGGACACTATTTGCGG	TGCTCGCGGACTCCAGATA	61.3℃	111
MSTRG.310247.4	NC_030836.1	TCTAACGAGATGGGGAGCCA	TCTAGCACCTAAGCATGGCG	59.4℃	198
MSTRG.310246.1	NC_030836.1	CATTGTCCAACCTAAAATAG	GGGGAATTTTGGTGGTCCAG	62.5℃	109
MSTRG.196987.6	NC_030822.1	ACGTCTGTACCCGAGTGTTTC	AGCGTTAGGAGACTGGAGGT	56.9℃	137
MSTRG.201344.1	NC_030822.1	AGTCCATGAACCACGCTCTG	CTGGTAGGCACTGCGAGTAG	63.0℃	161
UCP1	XM_018061376.1	CAAGATCTCAGCGGGCCTAA	TGTGCTTGCAGTCTGACCTT	62.3℃	87
β-actin	NM_001314342.1	CCTGCGGCATTCACGAAACTAC	ACAGCACCGTGTTGGCGTAGAG	59.7℃	87
